# Cross-national disparities in non-communicable disease: a universal health coverage-based service coverage index perspective, 2000–2021

**DOI:** 10.3389/fpubh.2026.1756485

**Published:** 2026-02-10

**Authors:** Mingzhu Zhou, Ying Jiang, Jiaying Zhu, Zhiyong Li, Hong Sun, Fangfang Gao, Kaiyuan Weng

**Affiliations:** 1 School of Pharmaceutical Business, Guangdong Pharmaceutical University, Guangzhou Higher Education Mega Center, Guangzhou, Guangdong, China; 2 College of Physical Education and Health, Chongqing College of International Business and Economics, Chongqing, China; 3 Shenzhen Nanshan Hospital of Chinese Medicine (Nanshan Hospital, The First Affiliated Hospital of Guangzhou University of Chinese Medicine), Shenzhen, Guangdong, China; 4 Medicine & Health Science College, Guangzhou Huashang College, Guangzhou, Guangdong, China

**Keywords:** cross-national disparities, inequality, NCDS, SCI, UHC

## Abstract

**Background:**

Universal Health Coverage (UHC) has garnered widespread attention since its inception. However, systematic research on the dynamic inequalities in UHC for Non-Communicable Diseases (NCDs) remains limited.

**Methods:**

This study investigates the inequalities in UHC for NCDs from 2000 to 2021, offering evidence and policy recommendations to promote global UHC equality for NCDs. Using multiple cross-national datasets, this study examines UHC inequality for NCDs. A panel fixed-effects model analyzes influencing factors, while the Gini coefficient and other indices measure inequality. The Oaxaca-Blinder decomposition method identifies sources of inequality differences among countries with varying income levels.

**Results:**

During this period, the global Non-Communicable Diseases Service Coverage Index (NCD-SCI) increased from the 50s to the 60s, while the Gini coefficient decreased from 0.115 to 0.095. Government health expenditure as a percentage of GDP significantly positively impacted NCD-SCI (*β* = 0.24, *p* < 0.01), whereas physician density per 10,000 people did not (95% CI included 0). The interaction between resource endowment and return rate contributed up to 53.26% to the SCI gap compared to low-income countries. Higher national income reduced the contribution of resource endowment differences to the SCI gap while increasing interaction effects. Differences between high-income and upper-middle-income countries were mainly due to health expenditure (contribution rate 116.67%), while those with low-income countries were driven by medical resource endowment.

**Conclusion:**

This study reveals long-term trends in UHC for NCDs amid global economic disparities and proposes measures to enhance UHC levels and promote equality in different regions.

## Introduction

Universal Health Coverage (UHC) (1), a pivotal target within the 2030 Agenda for Sustainable Development Goals (SDGs) (2), represents a shared objective for national governments and international organizations worldwide. Its vision aims to ensure that at least 80% of the global population gains access to quality health services by 2030 (3–5). Concurrently, it is committed to addressing health inequities—both within and between countries—through concerted efforts by governments and international bodies (2).

To quantify progress in UHC service coverage, the World Health Organization (WHO) and the World Bank jointly introduced the Service Coverage Index (SCI) in 2015 (6). The SCI utilizes 14 core indicators across four major service coverage domains—reproductive, maternal, newborn, and child health; infectious disease control; non-communicable diseases (NCDs); and service capacity and access—to measure member states’ advancement in delivering essential health services. This framework enables countries to identify gaps and inform policy interventions.

Particularly in recent years, NCDs—such as cardiovascular diseases, cancers, diabetes, and chronic respiratory diseases—have emerged as a priority focus within the context of UHC (7). Since the dawn of the 21st century, accelerated urbanization in lower middle-income countries (LMICs) (8, 9) has coincided with improved living standards. However, this progress has been counterbalanced by escalating NCD burdens in LMICs, attributable to widespread consumption of high-fat, high-salt, and high-sugar processed foods, reduced physical activity, and unhealthy cultural norms (10–12). These trends pose formidable challenges to individual health, household finances, and social development.

Currently, NCDs disproportionately contribute to the global disease burden, accounting for 73% of global deaths and 62% of the overall disease burden—a proportion that has risen continuously since 2000 (13). Although substantial progress in UHC was made over the past two decades, progress has stalled in recent years. Critically, since the inception of the Sustainable Development Goals era in 2015, the global SCI has increased by only three points (14). NCD management within UHC and primary healthcare (PHC) frameworks faces multifaceted challenges, characterized by: Limited resources, fragmented multi-sectoral PHC policies, inadequate financing, insufficient workforce capacity, incomplete essential service packages, ambiguous service delivery models, weak health information systems, barriers to diagnostic and medication access (15).

Additionally, under-investment in NCD research may exacerbate inequities in UHC across countries. In 2015, only 22% of countries had operational NCD research policies or programs; by 2019, approximately two-thirds of countries still lacked such frameworks, with a mere 4% of low-income countries adopting them (16). Consequently, NCD research priorities fail to fully align with population needs, and analyses of disparities within populations remain scarce. Concurrently, weak surveillance systems hinder effective NCD management. For instance, while the STEPS survey has been implemented in 120 countries, few nations repeat it every 5 years—as recommended by WHO (17). In 2019, only one-third of countries had robust cause-of-death mortality data systems, a disparity tightly correlated with national income groups: no low-income country possessed such systems, while over three-quarters of high-income countries did (18).

To address these gaps, this study leverages multiple transnational datasets to dissect global UHC performance for NCDs and its determinants. It compares disparities across income-based country groupings and proposes targeted recommendations, aiming to advance equitable UHC for NCDs worldwide.

## Method

### Data source

This study assessed global inequalities in the NCD-SCI from 2000 to 2021 using multiple cross-national datasets under the UHC framework. The primary data sources were:

1. NCD-SCI: Data from the WHO’s Universal Health Coverage Monitoring Database, covering essential NCD interventions (hypertension treatment, diabetes management, and cancer screening) with a score range of 0–100. Observations were used for 2000, 2005, 2010, 2015, 2017, 2019, and 2021. (The raw data were obtained from: https://www.who.int/data/gho/data/major-themes/universal-health-coverage-major).
2. Government Health Expenditure: Percentage of GDP data from the World Bank (2000–2021), based on WHO’s Global Health Expenditure Database (GHED). (The raw data were obtained from: https://data.worldbank.org/topic/health).
3. Physicians per 10,000 Population: Data (2000–2021) from WHO’s Global Health Workforce Statistics Database. (The raw data were obtained from: https://www.who.int/data/gho/data/themes/topics/health-workforce).
4. World Bank Income Classification: Country economic groupings based on GNI and inflation-adjusted thresholds. The 2025 classification was used to match data from 2000 to 2021. (The raw data were obtained from: https://datahelpdesk.worldbank.org/knowledgebase/articles/906519-world-bank-country-and-lending-groups).
5. GBD Database: NCD incidence, prevalence, DALYs, and mortality data (2021) from IHME’s GBD 2021 database, including sex-specific and combined estimates with 95% uncertainty intervals (UIs). (The raw data were obtained from: https://vizhub.healthdata.org/gbd-results/).

### Data harmonization and processing

All datasets were merged using ISO Alpha-3 country codes, with manual verification to resolve inconsistencies. Multilateral datasets were consolidated by ISO Alpha-3 country codes after manual verification and resolution of naming inconsistencies. Country income groups follow World Bank classifications throughout. Economies lacking any SCI value were excluded from the outset. Missing physician-density observations were imputed within each country via linear interpolation using a custom safe approx routine in R; remaining gaps were replaced with the country-specific long-term mean. For government health-expenditure-to-GDP ratios, missing values were first interpolated within country. If interpolation was impossible, the following hierarchical imputation sequence was applied: (i) country mean, (ii) year-specific global mean, (iii) overall grand mean. The final analytical sample consists of 190 economies with complete SCI records and assigned income-group information.

### Analysis of NCD burden

The study analyzed trends in NCD incidence, prevalence, DALYs, and mortality rates (including age-standardized rates, ASRs) globally from 2000 to 2021. Annual percentage changes (EAPCs) were calculated using a linear regression model *y* = *α* + *β*x + *ε*, where *y* = ln(ASR), *x* is the calendar year, and *β* represents the ASR trend. EAPC was derived as (exp(*β*) − 1) × 100%, with 95% confidence intervals (CIs) from the model. Trends were classified as increasing, decreasing, or stable based on EAPC and CI bounds (19, 20).

### Panel fixed-effects model analysis of the NCD-SCI

A panel fixed-effects model (21) was constructed to examine factors influencing NCD-SCI and its dynamic characteristics. The base model controlled for time trends and individual heterogeneity:

$$\begin{array}{c} \mathrm{sci}\_\mathrm{st}d_{\mathrm{it}}=\alpha +\beta_{1}\text{doctors}\_\mathrm{per}10k\_\mathrm{st}d_{\mathrm{it}}\\ +\beta_{2}\text{health}\_\exp\_\mathrm{gdp}\_\mathrm{st}d_{\mathrm{it}}+\gamma_{t}+\varepsilon_{\mathrm{it}} \end{array}$$

where i and t denote country and year, respectively; α is the constant term; β *_1_* and β _2_ represent the coefficients of the number of physicians per 10,000 population and the percentage of health expenditure in GDP on the standardized SCI value, respectively; $\gamma_{t}\;$ is the time fixed effect, which controls for common factors affecting all countries at different time points; and $\epsilon_{\mathrm{it}}$ is the error term.

The model underwent multicollinearity tests, heteroscedasticity tests, autocorrelation tests, cross-sectional dependence tests, and RESET tests to ensure the robustness and reliability of the results.

### Analysis of global inequality in NCD-SCI

Using balanced panel data (2000–2021), the study analyzed global inequalities in NCD-SCI through Gini coefficients ( $G=\frac{1}{2\mu n^{2}}\sum_{i=1}^{n}\sum_{j=1}^{n}|x_{i}-x_{j}|$ , G ranges from [0, 1], with values closer to 1 indicating a higher degree of inequality. $x_{i}$ represents the SCI value of country i, *μ* denotes the global mean of SCI, and *n* is the number of countries) (22). To assess the trends in inequality as measured by the Gini coefficient, we employed the Theil index and the Atkinson index. Theil indices $(T=\sum_{i=1}^{N}s_{i}(\frac{x_{i}}{\mu }\ln\frac{x_{i}}{\mu })),$ where i denotes the population weight of country i and Atkinson indices which is sensitive to the degree of inequality aversion, is also utilized to provide a comprehensive evaluation of inequality trends (23, 24). The Gini coefficient measured relative inequality, while absolute inequality was assessed via the P90–P10 gap. Inequalities were decomposed using the Oaxaca-Blinder method within a static panel fixed-effects model, with high-income countries as the baseline. Bootstrap sampling (1,000 replications) validated the results. Typical country cases within each income group were identified to highlight key inequality features.

All statistical analyses in this study were completed using R software (version 4.3.2).

## Results

### Analysis of NCD burden

**Figure 1:**
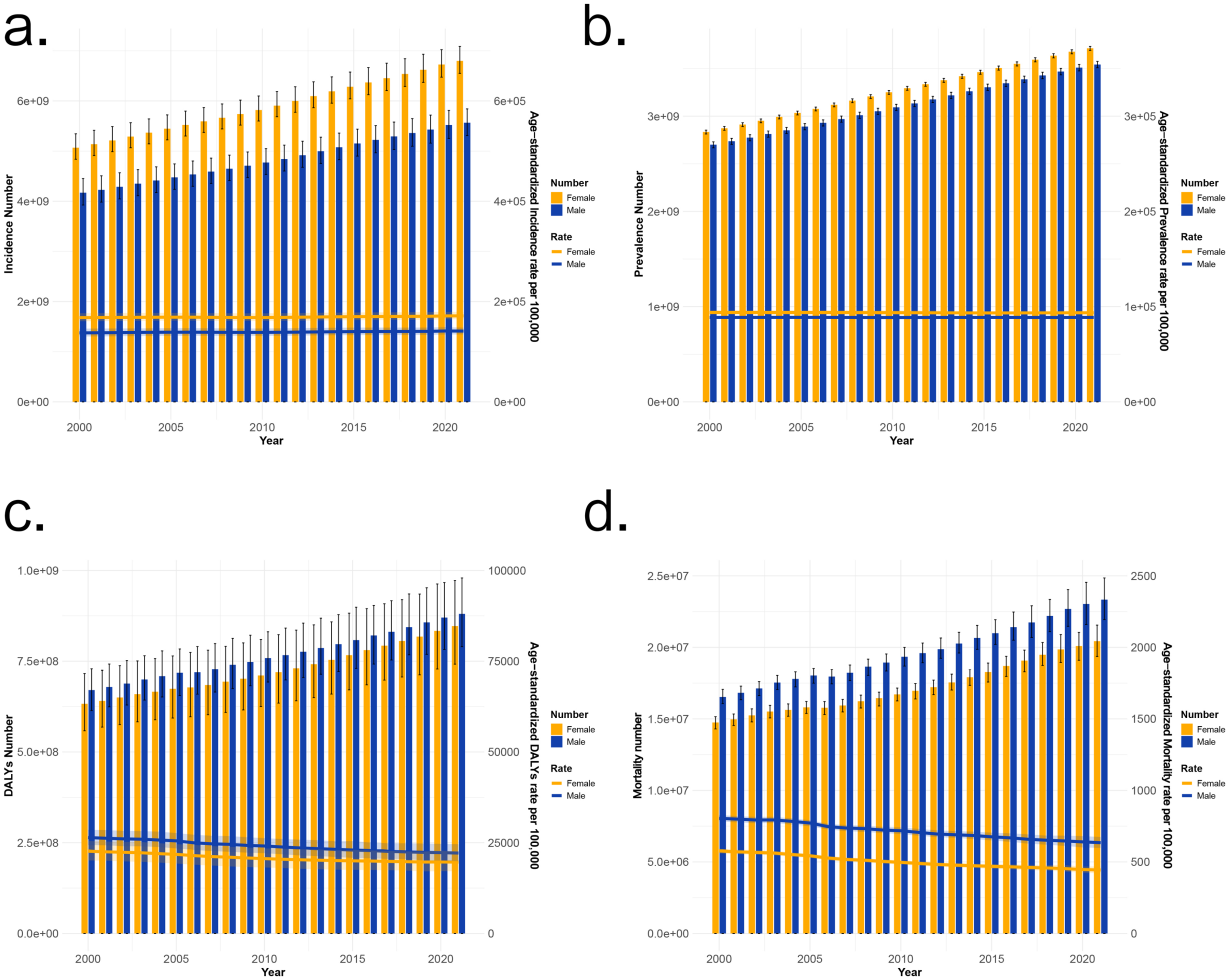
2000–2021 Non-communicable diseases’ trend. The number and ASR of incidence **(a)**, prevalence **(b)** DALYs **(c)**, and mortality **(d)** in 2000–2021 Non-communicable diseases’ trend.

Supplementary Tables S1–S4 displays the counts and age-standardized rates (ASRs) of incidence, prevalence, disability-adjusted life years (DALYs), and mortality for non-communicable diseases (NCDs) from 2000 to 2021. With 2000 as the baseline, it is observed that the burden of NCDs is gradually increasing year by year. Among them, although the standardized incidence and prevalence rates in females are higher than those in males, the age-standardized disability-adjusted life years rate (ASDR) and age-standardized mortality rate (ASMR) are much higher in males than in females (Figure 1). At the global level, the overall control of NCDs is good, with both ASDR and ASMR showing a downward trend. At the regional level, the East Asia and Pacific region has the highest increase in the age-standardized incidence rate (ASIR) of NCDs at 0.12%, while the Middle East and South Africa region has the largest decrease in ASIR at 0.04%. The age-standardized prevalence rate (ASPR) in the Europe and Central Asia region tends to be stable. The Europe and Central Asia region also has the highest decrease in ASDR and ASMR, at 1.25 and 1.87%, respectively. In contrast, South Asia has the lowest decrease in ASDR and ASMR, at 0.33 and 0.12%, respectively.

### Global panel fixed-effects model analysis of NCDs

**Figure 2:**
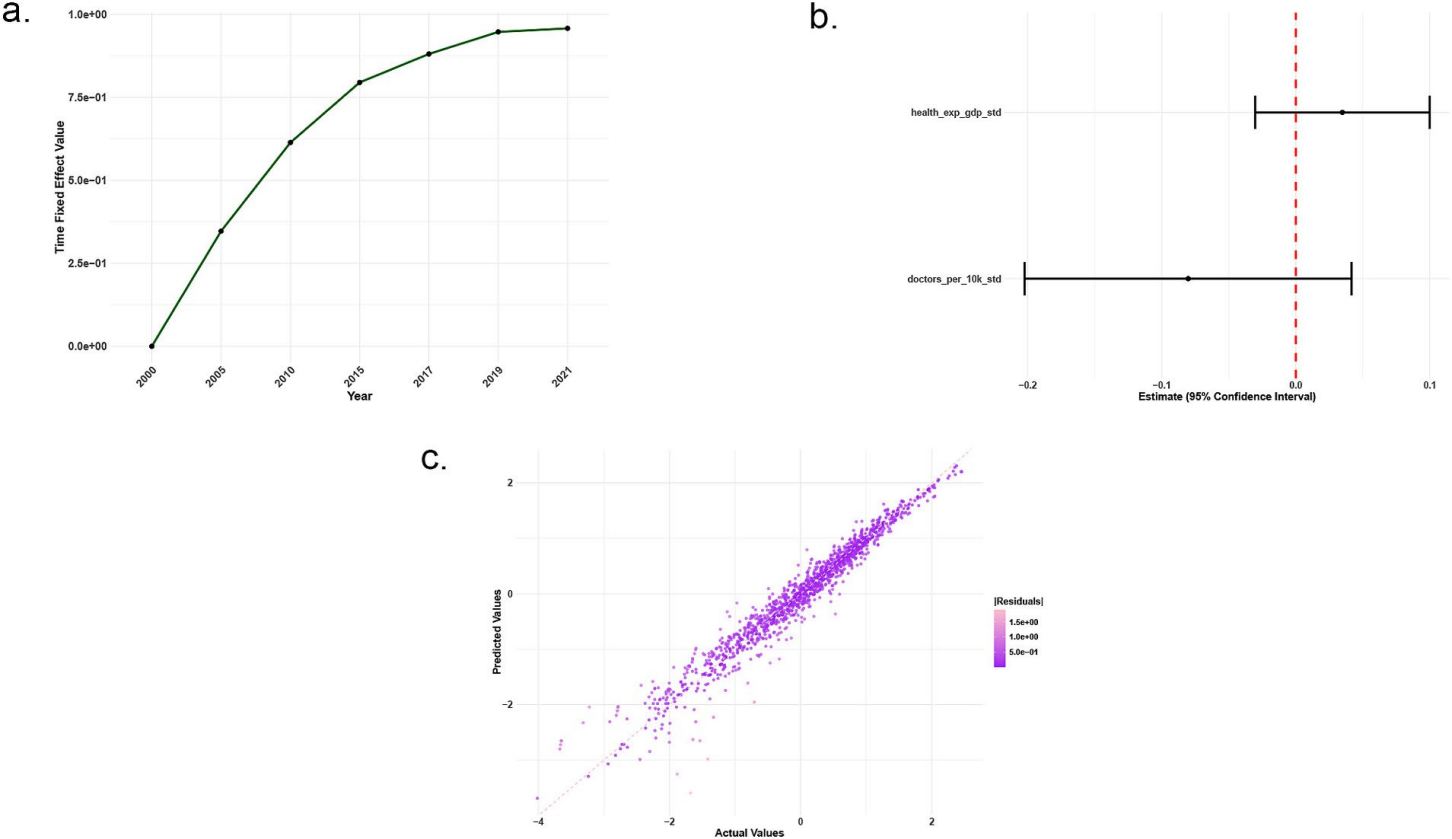
Basic two-way fixed effects model. Trend chart of time fixed effects values **(a)**, coefficient estimates **(b)**, scatter plot of actual vs. predicted values **(c)**.

Figure 2a shows that time fixed-effect values increase over time, indicating a significant positive impact of year on SCI during the study period, likely reflecting global progress in NCD prevention and control. Figure 2b reveals that the coefficient estimate for physicians per 10,000 population is not statistically significant (95% CI includes 0), while health expenditure as a percentage of GDP has a positive impact on SCI. Figure 2c indicates a good model fit, with points closely distributed near the 45-degree diagonal and small residuals.

Model validation results are in Supplementary Figure S1.

## Analysis of international inequality in the NCD-SCI

### Descriptive analysis of NCD-SCI

**Figure 3:**
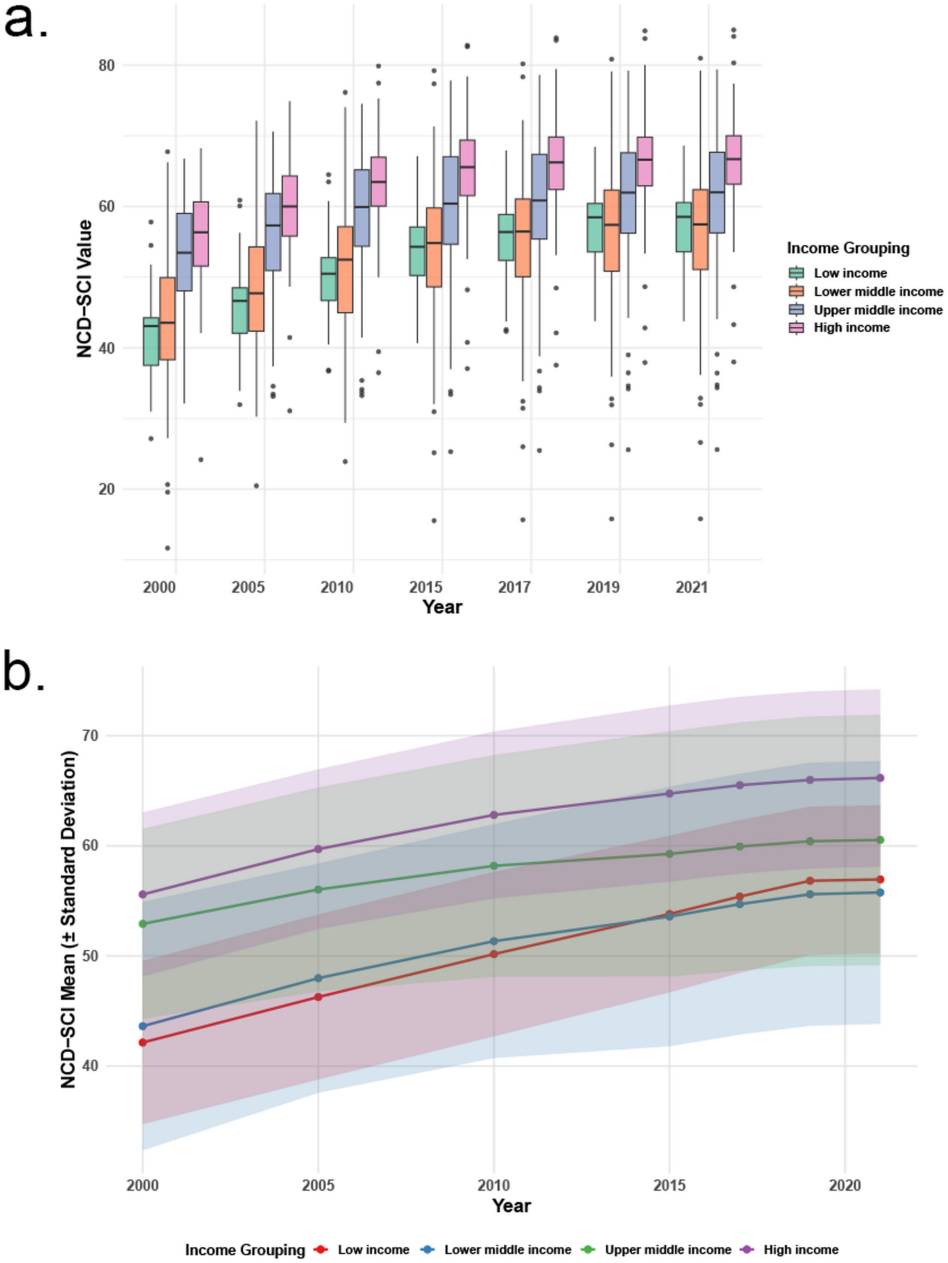
2000–2021, Global NCD-SCI trend. Changes in the distribution of NCD-SCI values by income group from 2000 to 2021 **(a)**, time-dependent dynamics of NCD-SCI mean and standard deviation across income groups from 2000 to 2021 **(b)**.

After matching the World Bank income groups with the NCD- SCI data provided by the World Health Organization, it was observed that the global distribution gap in universal health coverage among low-income countries has gradually narrowed compared to lower-middle-income countries and even showed a trend of surpassing the coverage rate of lower-middle-income countries in 2015. In contrast, although the universal health coverage rate in high-income countries has been increasing year by year, the overall room for improvement is limited, and a plateau phase even began in 2015 (see Figure 3) (The relevant test are shown in Supplementary Table S5).

### Analysis of global inequality trends in NCD-SCI

**Figure 4:**
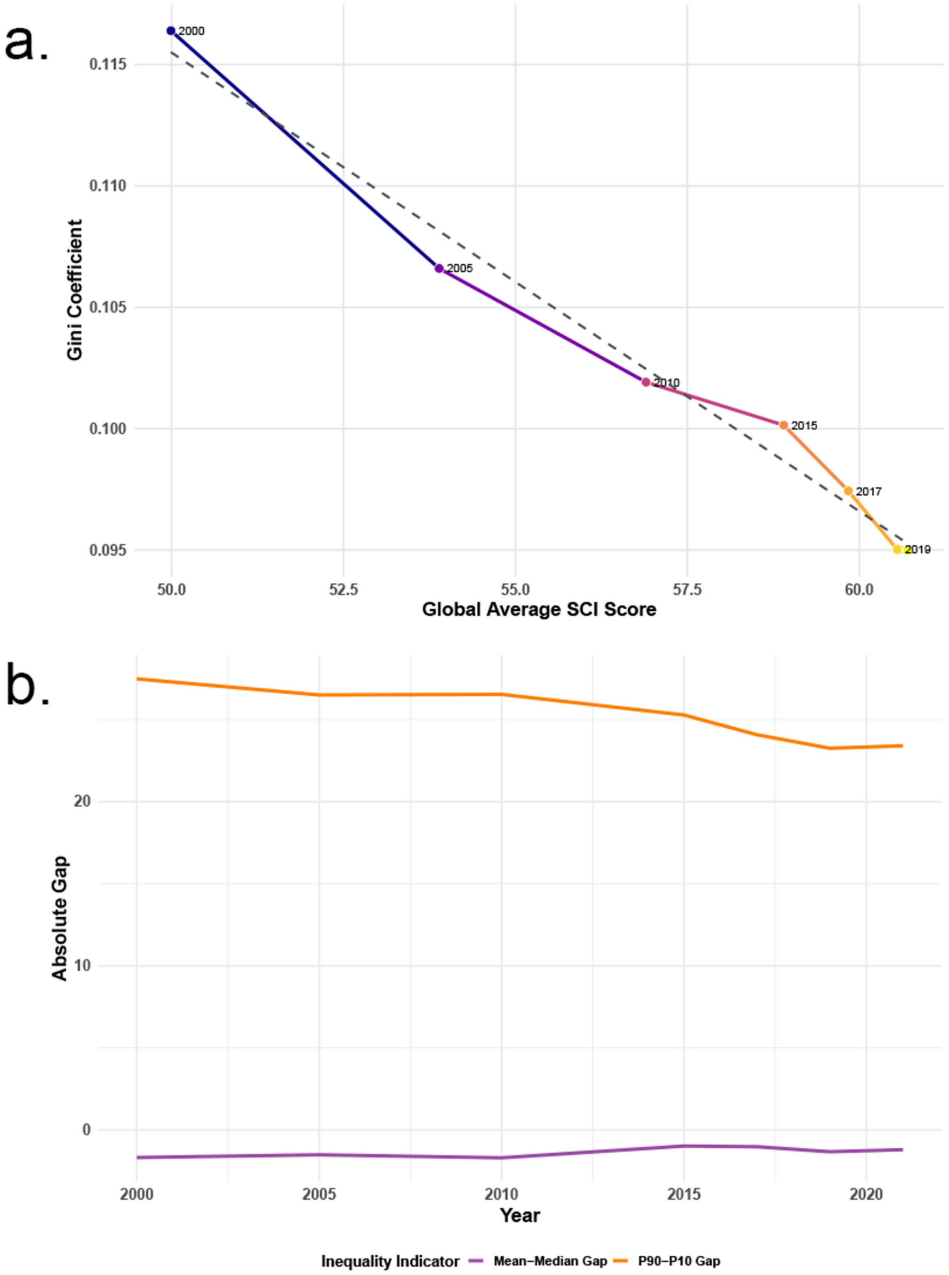
2000–2021, Global NCD-SCI inequality trend. Gini coefficient vs. global average SCI score **(a)**, absolute gap over time by inequality indicator **(b)**.

The global average NCD-SCI level rose from 50s to 60s, while the Gini coefficient decreased from 0.115 to 0.095, indicating a significant negative correlation between them. From 2000 to 2019, the degree of global NCD-SCI inequality improved, with narrowing disparities among countries (Figure 4a). The Theil index (<0.03) and Atkinson index (*ε* = 0.5, 0.15) both indicated mild inequality, supporting this finding. Future analyses should focus on a few extremely low-income countries and potential internal inequalities masked by policy (Supplementary Figure S2). From 2000 to 2021, global absolute NCD-SCI inequality showed varied trends across dimensions. The gap between the mean and median remained stable, suggesting no significant changes in the distribution structure of universal health coverage. The difference between the 90th and 10th percentiles was high in 2000 and gradually declined, stabilizing around 2020 (Figure 4b).

### Oaxaca-blinder decomposition of inequality by income group based on panel fixed-effects model

**Figure 5:**
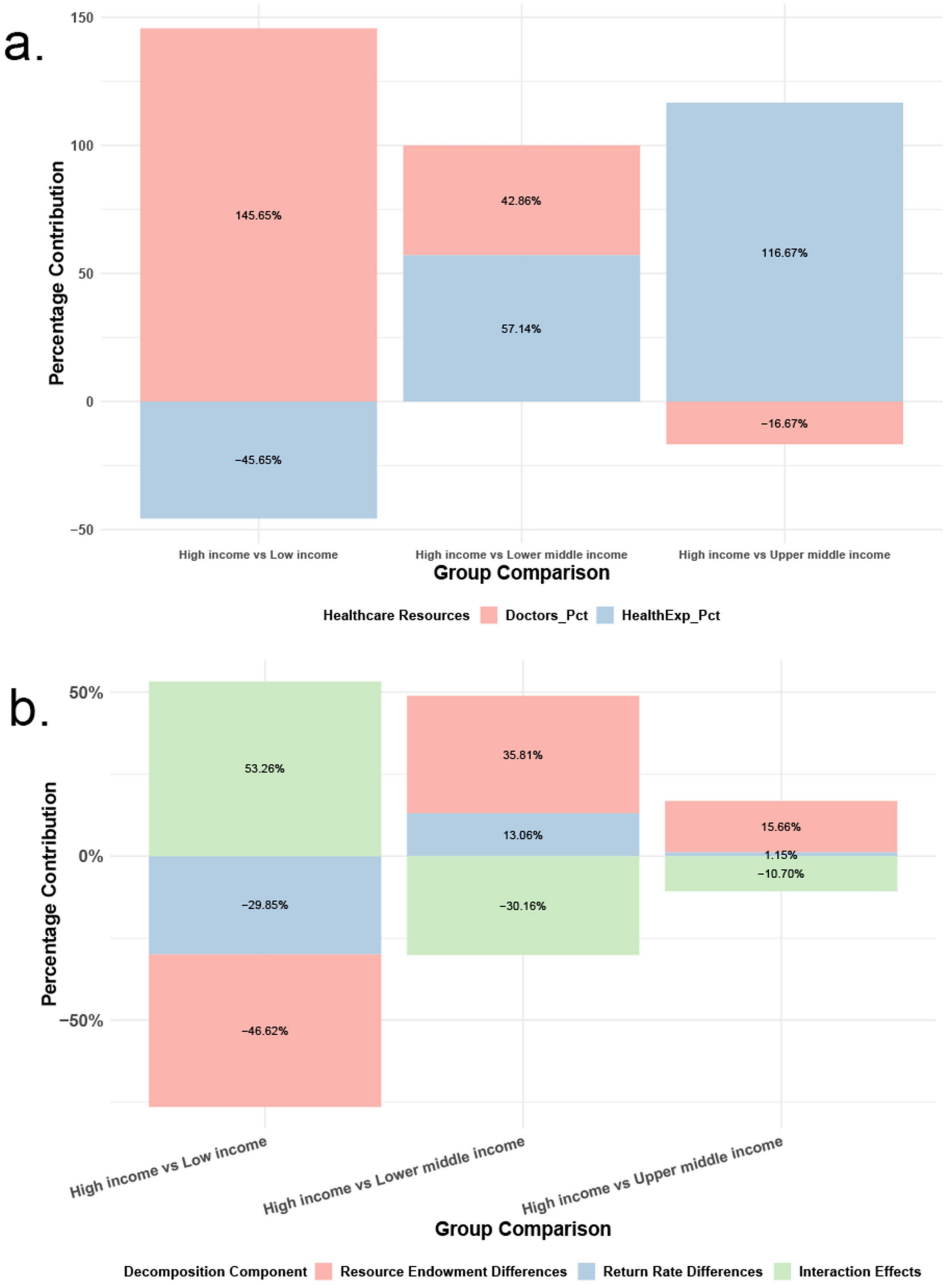
Oaxaca-Blinder decomposition. Percentage contribution of SCI disparities across different income groups **(a)**, decomposition of SCI disparities **(b)**.

To investigate factors influencing NCD-SCI inequality, this study used a panel fixed-effects model to decompose inequality by income group. Compared with high-income countries, low-income countries had a 145.65% contribution of medical resource difference (physicians per 10,000 population) to the SCI gap, while health expenditure difference contributed −45.65%, narrowing the gap. Lower-middle-income countries had a 42.86% contribution from medical resources and a 57.14% contribution from health expenditure, indicating significant physician shortages and a more pronounced impact of health expenditure insufficiency. Upper-middle-income countries showed a −16.67% contribution from medical resources, suggesting a minor impact on SCI, and a 116.67% contribution from health expenditure, highlighting the most significant impact of health expenditure insufficiency on SCI (Figure 5a).

Based on the aforementioned results, this study further decomposed the SCI gap. As shown in Figure 5b, when comparing high-income countries with low-income countries, the interaction effect contributed the most to the SCI gap at 53.26%, indicating that the interplay between resource endowment and the rate of return significantly exacerbated the gap between low-income and high-income countries. The difference in resource endowment contributed −46.62% to the SCI gap, suggesting that the overall shortage of resources in low-income countries is one of the main reasons for the SCI gap. When comparing high-income countries with lower-middle-income countries, the difference in resource endowment contributed the most to the SCI gap at 35.81%, followed by the interaction effect at −30.16%. When comparing high-income countries with upper-middle-income countries, the difference in resource endowment again contributed the most to the SCI gap at 15.66%, followed by the interaction effect at −10.70%, while the difference in the rate of return contributed the least at 1.15%.

Nevertheless, according to Supplementary Tables S6–S8, the difference in resource endowment was not significant between high-income and low-income countries, or between high-income and lower-middle-income countries, but it was significant between high-income and upper-middle-income countries, indicating that the resource level of the upper-middle-income group is closer to that of the high-income group. In terms of the difference in the rate of return and the interaction effect, the confidence intervals all included zero, and the differences between all groups were not significant. Overall, the differences between groups were mainly caused by the difference in resource endowment, especially between the high-income and upper-middle-income groups, while other influencing factors were relatively minor. For detailed data, please refer to Supplementary Tables S6–S8.

### Analysis of typical countries

**Figure 6:**
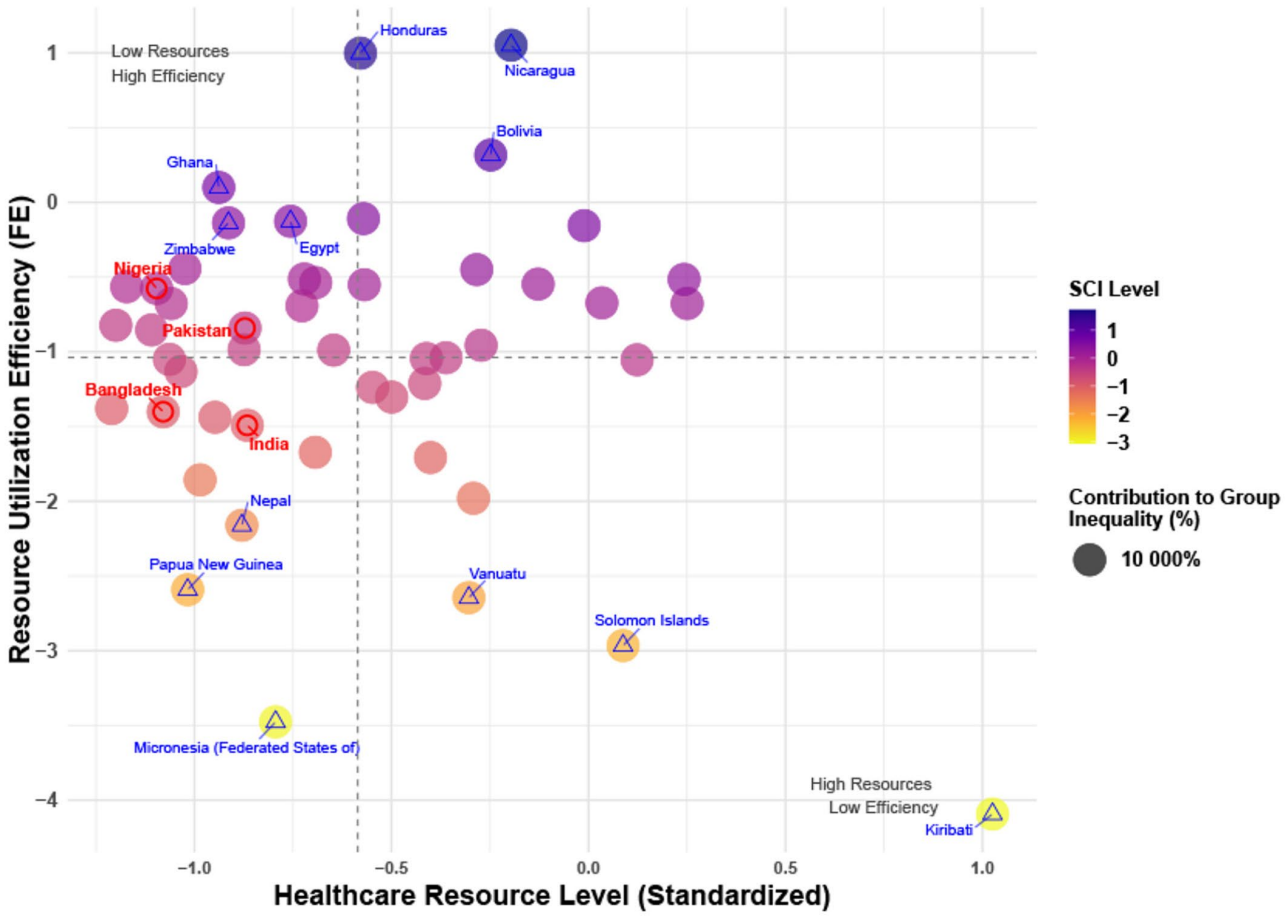
Healthcare resources and efficiency quadrant.

For countries with high levels of inequality as indicated by Gini coefficient comparisons, further analysis revealed that Sierra Leone, Micronesia, Tonga, and Nauru were identified as the most unequal countries in the low-income, lower-middle-income, upper-middle-income, and high-income groups, respectively (Supplementary Tables S9, S10). Despite their limited resources, countries such as Ghana, Nigeria, and Pakistan demonstrated relatively high resource utilization efficiency. Conversely, Nicaragua, Bolivia, and Colombia, despite having adequate resources, exhibited relatively low resource utilization efficiency. Moreover, some countries, such as Nepal and Micronesia, were found to be at a double disadvantage in terms of both resource availability and utilization efficiency (see Figure 6).

## Discussion

This study systematically evaluates disparities in NCD burden and SCI within the framework of universal health coverage, utilizing comprehensive data spanning global, regional, and national scales from 2000 to 2021.

The findings indicate that the global NCD burden remained largely stabilized, with health inequalities showing improvement prior to the COVID-19 pandemic, while a modest resurgence in inequality trends was observed in the post-pandemic period (25). Regarding disease burden distribution, males bore a disproportionately higher burden of mortality and disability. Regionally, Europe and Central Asia achieved substantial declines in age-standardized death rates (ASDR), attributable to well-established healthcare systems and greater investment. Conversely, South Asia, East Asia, and the Pacific exhibited more pronounced increases in age-standardized incidence rates (ASIR), driven by rapid urbanization and lifestyle transitions, thus warranting enhanced control measures (26, 27).

Meanwhile, few studies have conducted modeling analyses on the SCI index, within the context of UHC. Globally, the SCI index has shown an upward trend over time, which may be associated with improvements in the global public health environment. Government health expenditure as a percentage of GDP exerts a significant positive impact on the SCI index, indicating that increasing health expenditure can effectively enhance the service coverage level for NCDs. In contrast, the impact of primary healthcare resources (i.e., the number of physicians per 10,000 population) is not significant, as observed in countries such as South Korea, Cyprus, Costa Rica, and Peru. The performance of these countries in terms of effective coverage varies substantially due to differences in per capita pooled health expenditure, highlighting that while increasing health expenditure is necessary, it alone is insufficient to improve the effective coverage rate of UHC. In other words, the efficiency of resource utilization may be more crucial than the total volume of resources. For instance, countries with limited resources, such as Ghana and Nigeria, have achieved better SCI performance than resource-abundant but inefficient countries like Nicaragua and Bolivia. This also explains why some countries have failed to effectively elevate NCD service coverage despite merely expanding the number of physicians (28, 29).

From the perspective of the developmental trend in coverage level, the UHC pertaining to NCDs has maintained a steady upward trajectory, while exhibiting notable disparities across income groups. Following the launch of the Sustainable Development Goals (SDGs) in 2015, low-income countries registered accelerated growth in this regard with international assistance. This phenomenon is closely associated with the financial input and priority focus areas during the SDG era and the Millennium Development Goals (MDGs) era (30). In particular, under the framework of the MDGs, sub-Saharan Africa secured increased financial support and policy preferences for HIV prevention and control, vaccination [e.g., measles vaccine and diphtheria-tetanus-pertussis (DPT) vaccine], the treatment of childhood infectious diseases (e.g., respiratory tract infections and diarrheal diseases), and maternal health services. The service coverage in these domains has been significantly improved, thereby accelerating the enhancement of the effective coverage index of UHC in sub-Saharan Africa (31) (with a year-on-year growth rate of 2.6% as of 2019).

A subgroup comparison using high-income countries as the baseline revealed that upper-middle-income countries have a relatively narrow gap with high-income countries in terms of resource endowments, and thus should prioritize targeted interventions (32). For instance, Peru has effectively improved the accessibility of primary healthcare services by increasing healthcare investment (a 15% growth in the healthcare budget in 2023, allocated to facility upgrading, personnel training, pharmaceutical procurement, and public health programs) and optimizing resource allocation (establishing special funds for rural healthcare and constructing new primary care clinics). In contrast, high-income countries have entered a developmental plateau with limited room for further improvement, as their NCD-SCI has already reached a high level (33, 34). Among them, the Republic of Korea has continuously optimized healthcare coverage through measures including the establishment of a nationwide unified healthcare insurance system, the refinement of the cost-sharing mechanism, and the setup of the National Evidence-based Healthcare Collaboration Agency (NECA). These efforts have provided mature experience for global practices in UHC (35).

It is worth noting that among the 17.3 million global NCD-related deaths in 2021, 86% of premature deaths occurred in lower-middle-income countries, whereas high-income countries have reaped the greatest benefits from the current pattern. This not only indicates that sustained expenditure and flexible resource allocation are pivotal to maintaining coverage equity, but also reveals the challenges inherent in achieving absolute equity (36). It follows that strategies should be tailored to the developmental stages of different countries: upper-middle-income countries (e.g., Peru) need to implement targeted interventions and optimize investment and resource distribution; high-income countries (e.g., the Republic of Korea) are expected to drive systemic innovations to provide mature experience for global practices (28, 29); low- and lower-middle-income countries, in turn, are in urgent need of a dual impetus from policy reform and technological innovation, so as to expand resource provision while improving efficiency, thereby narrowing the existing gaps. Specifically, targeted policies can be formulated to address the respective weaknesses of each country. For example, Viet Nam has strived to bridge urban–rural and regional resource gaps (37); Saudi Arabia has strengthened health interventions targeting populations with low educational attainment (38); and Sudan has developed tele-medicine services to ensure healthcare service continuity (39).

Going forward, to advance health equity, the WHO and countries worldwide must prioritize the issue of unequal resource allocation for NCDs. The advancement of the WHO Pandemic Agreement (WHOPA) (40) provides new opportunities for multilateral cooperation, developed countries should support recipient nations in enhancing their health technology capacity, while international organizations can focus on addressing service accessibility issues caused by geographical barriers through aid programs. The global community should collectively commit to optimizing resource allocation policies and implementing the One Health concept (41).

## Conclusion

In conclusion, this study confirms that although NCD service coverage is constrained by multiple factors, targeted public health policy interventions are crucial. The core focus moving forward should be shifting from mere increases in investment to improving resource utilization efficiency, and leveraging international cooperation to target effective interventions at the most vulnerable regions and populations.

## Data Availability

The datasets presented in this study can be found in online repositories. The names of the repository/repositories and accession number(s) can be found in the article/Supplementary material.

## Glossary

ASDR: Age-standardized disability-adjusted life years rate
ASIR: Age-standardized incidence rate
ASMR: Age-standardized mortality rate
ASPR: Age-standardized prevalence rate
ASR: Age-standardized rate
CI: Confidence interval
DALYs: Disability-adjusted life years
DTP: Diphtheria-tetanus-pertussis
EAPC: Estimated annual percentage change
GBD: Global burden of disease
GHED: Global health expenditure database
GNI: Gross national income
IHME: Institute for Health Metrics and Evaluation
LMICs: Lower middle-income countries
MDGs: Millennium development goals
NCD-SCI: Non-Communicable diseases service coverage index
NCDs: Non-Communicable diseases
NECA: National evidence-based healthcare collaboration agency
NSFC: National natural science foundation of china
PHC: Primary health care
SDGs: Sustainable development goals
SDI: Socio-demographic index
SCI: Service coverage index
STEPS: STEP wise approach to surveillance (WHO)
UHC: Universal health coverage
UIs: Uncertainty intervals
WB: World Bank
WHO: World Health Organization
WHOPA: WHO Pandemic Agreement
