# Correction: Cross-national disparities in non-communicable disease: a universal health coverage-based service coverage index perspective, 2000–2021

**DOI:** 10.3389/fpubh.2026.1817661

**Published:** 2026-03-10

**Authors:** Frontiers Production Office

**Affiliations:** Frontiers Media SA, Lausanne, Switzerland

**Keywords:** cross-national disparities, inequality, NCDS, SCI, UHC

There was a mistake in Figure 1 as published. The right-hand *Y*-axis labels for panels **(a)**, **(b)**, and **(d)** showing “Age-standardized Incidence rate per 100,000”, “Age-standardized Prevalence rate per 100,000” and “Age-standardized Mortality rate per 100,000” were erroneously denoted as “Age-standardized DALYs rate per 100,000” due to a typographical oversight. The corrected Figure 1 appears below.

**Figure 1:**
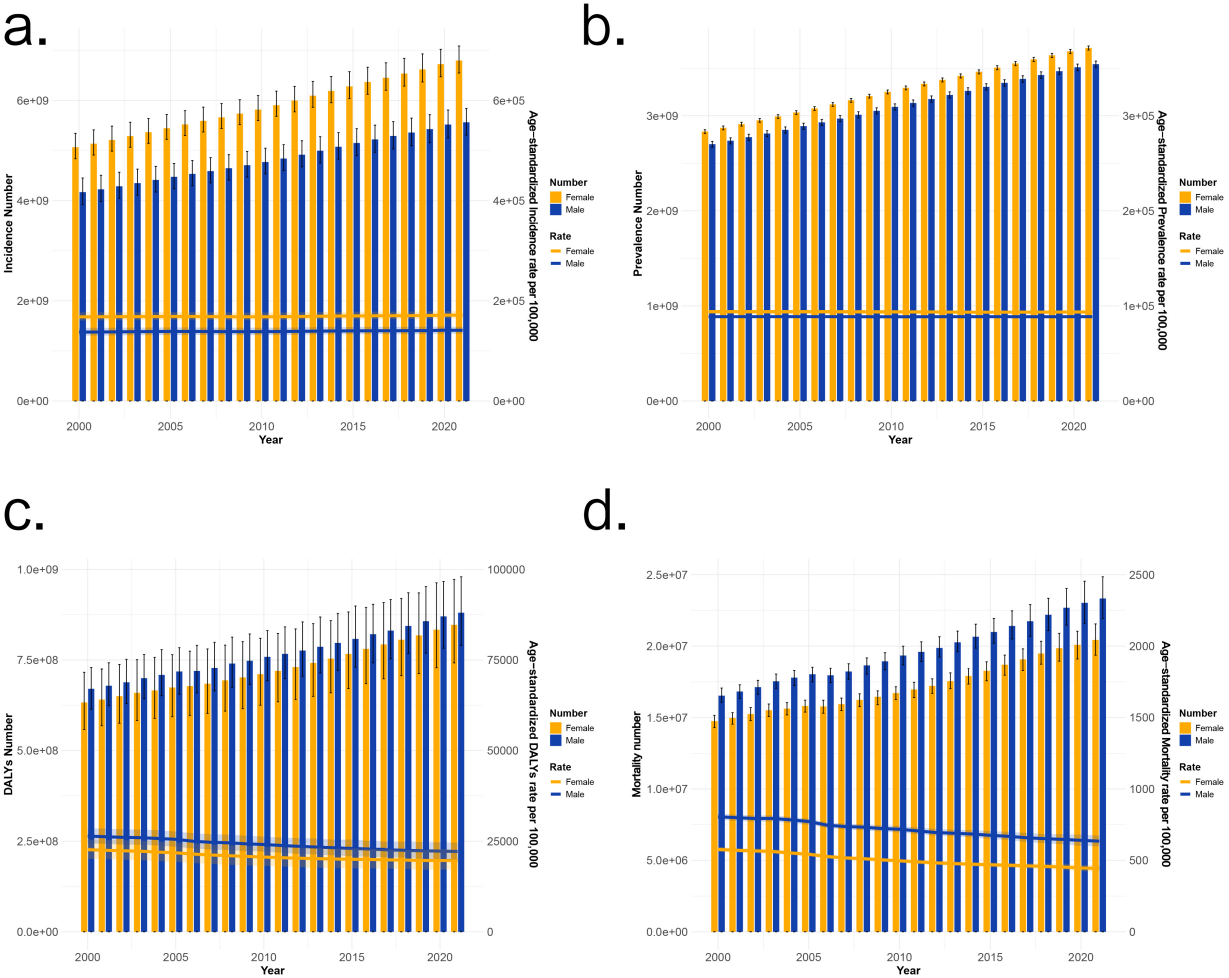
2000–2021 Non-communicable diseases' trend. The number and ASR of incidence **(a)**, prevalence **(b)** DALYs **(c)**, and mortality **(d)** in 2000–2021 Non-communicable diseases' trend.

In the section **Results**, sub-section *Global panel fixed-effects model analysis of NCDs*, the authors had requested that the phrase “Figure 2b reveals that the coefficient estimate for physicians per 10,000 population is not statistically significant (95% CI includes 0), while health expenditure as a percentage of GDP has a significant positive impact on SCI (95% CI does not include 0).” should be revised to the more conservative statement: “while health expenditure as a percentage of GDP has a positive impact on SCI.” However, due to an oversight, the correction instruction itself was inadvertently inserted into the main text instead of implementing the actual revision.

A correction has been made to the section **Results**, sub-section *Global panel fixed-effects model analysis of NCDs* so that the sentence now reads:

“**Figure 2b** reveals that the coefficient estimate for physicians per 10,000 population is not statistically significant (95% CI includes 0), while health expenditure as a percentage of GDP has a positive impact on SCI.”

The original version of this article has been updated.

## Generative AI statement

Any alternative text (alt text) provided alongside figures in this article has been generated by Frontiers with the support of artificial intelligence and reasonable efforts have been made to ensure accuracy, including review by the authors wherever possible. If you identify any issues, please contact us.

