# Supplementary material for: Cross-national disparities in non-communicable disease: a universal health coverage-based service coverage index perspective, 2000–2021
Source: Front Public Health. 2026 Feb 10;14:1756485. doi: 10.3389/fpubh.2026.1756485 (PMC12929431; doi:10.3389/fpubh.2026.1756485)
Supplement: Supplementary file 1 [file Data_Sheet_1.docx]

**Supplementary Materials**

# Table S1. The case number and ASR of Incidence of NCDs in 2000 and 2021 for both sexes by Global and by World Bank regions.

| location | 2000 | | 2021 | | EAPC(95% CI)  1990-2021 |
| --- | --- | --- | --- | --- | --- |
|  | Number(95%UIs) | ASR(95%UIs) | Number(95%UIs) | ASR(95%UIs) |  |
| Global | 9238052895 (8775389272 to 9784955028) | 152723.27 (145444.68 to 160961.76) | 12364222888 (11859103110 to 12922829056) | 156214.82 (149373.11 to 163580.88) | 0.1 (0.08 to 0.12) |
| East Asia & Pacific - WB | 2695841088 (2554948968 to 2875999854) | 133349.4 (126346.73 to 141979.65) | 3297295098 (3154430461 to 3473097856) | 138334.19 (130838.37 to 146876.08) | 0.12 (0.07 to 0.16) |
| Europe & Central Asia - WB | 1403123634 (1349760390 to 1468106716) | 156040.75 (149029.06 to 164267.27) | 1529865340 (1475338115 to 1595647651) | 157033.3 (150316.69 to 165179.43) | 0.01 (-0.01 to 0.03) |
| Latin America & Caribbean - WB | 844660576 (799146491 to 896314199) | 165681.45 (157725.97 to 174671.82) | 1113941616 (1068846501 to 1166325696) | 167710.7 (160391.79 to 176259.66) | 0.02 (0 to 0.04) |
| Sub-Saharan Africa - WB | 1181186592 (1113425324 to 1247928388) | 180496.97 (172982.24 to 188611.01) | 2047310271 (1948818147 to 2147792383) | 179699.06 (172759.55 to 186714.22) | -0.01 (-0.03 to 0) |
| Middle East & North Africa - WB | 455862624 (429000566 to 489203190) | 146553.94 (139351.35 to 154859.47) | 686947463 (656885984 to 720953659) | 145123.74 (139216.87 to 151853.49) | -0.04 (-0.08 to 0.01) |
| South Asia - WB | 2208761405 (2081994689 to 2367230912) | 164689.84 (156899.99 to 173952.8) | 3122043593 (2991190228 to 3265058855) | 165305.93 (158659.72 to 172474.8) | 0.06 (0.02 to 0.11) |
| World Bank Regions | 9229663152 (8767357283 to 9776110766) | 152771.94 (145491.6 to 161011.99) | 12354266201 (11849542669 to 12912368754) | 156255.72 (149413.41 to 163622.84) | 0.1 (0.08 to 0.12) |

# Table S2. The case number and ASR of prevalence of NCDs in 2000 and 2021 for both sexes by Global and by World Bank regions.

| location | 2000 | | 2021 | | EAPC(95% CI)  1990-2021 |
| --- | --- | --- | --- | --- | --- |
|  | Number(95%UIs) | ASR(95%UIs) | Number(95%UIs) | ASR(95%UIs) |  |
| Global | 5535187283 (5484614989 to 5584677951) | 91157.24 (90370.26 to 91932.54) | 7255129087 (7201504217 to 7305480908) | 91033.98 (90296.74 to 91725.48) | -0.01 (-0.01 to -0.01) |
| East Asia & Pacific - WB | 1847948224 (1831438452 to 1864285530) | 89815.93 (88932.59 to 90710.55) | 2197430848 (2181554406 to 2212195803) | 89476.53 (88560.62 to 90344.98) | -0.03 (-0.03 to -0.02) |
| Europe & Central Asia - WB | 805439380 (799643300 to 810630760) | 89809.81 (88932.28 to 90595.1) | 860152551 (854344648 to 865309722) | 89714.91 (88787.14 to 90559.3) | 0 (-0.01 to 0) |
| Latin America & Caribbean - WB | 466991206 (461743551 to 472047880) | 90983.95 (90095.45 to 91851.82) | 607862540 (602638238 to 612632336) | 90764.49 (89873.9 to 91597.03) | -0.02 (-0.03 to -0.02) |
| Sub-Saharan Africa - WB | 609569436 (603288917 to 615603212) | 93617.28 (93009.66 to 94222.55) | 1058355299 (1047500870 to 1068178730) | 92754.27 (92104.63 to 93362.33) | -0.05 (-0.05 to -0.04) |
| Middle East & North Africa - WB | 285731002 (282195369 to 289190175) | 91714.14 (90827.14 to 92580.81) | 435317307 (431351641 to 439325427) | 91522.63 (90693.03 to 92361.8) | -0.02 (-0.02 to -0.01) |
| South Asia - WB | 1230053507 (1216256040 to 1243313419) | 92287.99 (91480.43 to 93061.07) | 1743094559 (1729014814 to 1757113395) | 91552.83 (90830.08 to 92272.02) | -0.04 (-0.05 to -0.04) |
| World Bank Regions | 5528356841 (5477847507 to 5577795153) | 91157.01 (90370.21 to 91932.14) | 7247290743 (7193719338 to 7297598928) | 91033.88 (90296.67 to 91725.43) | -0.01 (-0.01 to -0.01) |

# Table S3. The case number and ASR of DALYs of NCDs in 2000 and 2021 for both sexes by Global and by World Bank regions.

| location | 2000 | | 2021 | | EAPC(95% CI) 1990-2021 |
| --- | --- | --- | --- | --- | --- |
|  | Number(95%UIs) | ASR(95%UIs) | Number(95%UIs) | ASR(95%UIs) |  |
| Global | 1303111592 (1171880119 to 1448728461) | 24373.03 (22065.3 to 26914.76) | 1727188945 (1537884455 to 1941634668) | 20783 (18495.72 to 23367.89) | -0.82 (-0.87 to -0.77) |
| East Asia & Pacific - WB | 429462455 (387632859 to 473931269) | 23636.23 (21538.62 to 25866.6) | 563579087 (491944823 to 639847722) | 18985.66 (16584.82 to 21631.25) | -1.18 (-1.27 to -1.09) |
| Europe & Central Asia - WB | 261154214 (238110333 to 288384487) | 24397.08 (22102 to 27131.12) | 259560672 (232183409 to 291688069) | 19377.23 (17051.55 to 22201.15) | -1.25 (-1.33 to -1.17) |
| Latin America & Caribbean - WB | 96740424 (85368073 to 109852704) | 22591.57 (20206.59 to 25417.68) | 140331551 (123325006 to 161082927) | 20291.86 (17845.68 to 23315.45) | -0.55 (-0.59 to -0.5) |
| Sub-Saharan Africa - WB | 121068012 (107403441 to 135296028) | 26957.11 (24584.65 to 29764.91) | 181622231 (158599594 to 207876861) | 23910.04 (21301.69 to 27013.96) | -0.57 (-0.59 to -0.55) |
| Middle East & North Africa - WB | 59804650 (53240656 to 67304535) | 26647.89 (24194.23 to 29414.91) | 90657191 (78355159 to 105602115) | 23141.13 (20348.69 to 26528.42) | -0.7 (-0.73 to -0.68) |
| South Asia - WB | 250865356 (223402822 to 280491851) | 25188.49 (22661.48 to 27908.02) | 381022260 (334665991 to 432271169) | 23114.27 (20544.1 to 25964.11) | -0.33 (-0.37 to -0.3) |
| World Bank Regions | 1301217781 (1170159612 to 1446642273) | 24370.54 (22062.82 to 26912.27) | 1724981513 (1535857176 to 1939187418) | 20781.21 (18493.8 to 23366.15) | -0.82 (-0.87 to -0.77) |

# Table S4. The case number and ASR of Deaths of NCDs in 2000 and 2021 for both sexes by Global and by World Bank regions.

| location | 2000 | | 2021 | | EAPC(95% CI) 1990-2021 |
| --- | --- | --- | --- | --- | --- |
|  | Number(95%UIs) | ASR(95%UIs) | Number(95%UIs) | ASR(95%UIs) |  |
| Global | 31278953 (30608205 to 32035442) | 678.76 (664.78 to 694.19) | 43768182 (41841301 to 45882469) | 529.68 (506.58 to 554.52) | -1.24 (-1.3 to -1.18) |
| East Asia & Pacific - WB | 10888497 (10375588 to 11424736) | 716.72 (685.79 to 748.76) | 16247438 (14639144 to 17926290) | 524.46 (474.49 to 576.93) | -1.68 (-1.79 to -1.57) |
| Europe & Central Asia - WB | 8025220 (7986953 to 8073484) | 682.61 (679.11 to 686.94) | 8212712 (7976793 to 8454480) | 471.81 (457.4 to 486.38) | -1.87 (-1.95 to -1.78) |
| Latin America & Caribbean - WB | 1957316 (1937320 to 1978068) | 555.88 (550.69 to 561.79) | 3124983 (2970228 to 3304034) | 455 (432.23 to 481.18) | -0.9 (-0.96 to -0.84) |
| Sub-Saharan Africa - WB | 2118988 (1974654 to 2258242) | 747.98 (699.68 to 794.26) | 3061759 (2739373 to 3387658) | 647.85 (591.05 to 706.55) | -0.67 (-0.69 to -0.65) |
| Middle East & North Africa - WB | 1127324 (1082387 to 1177523) | 761.88 (736.67 to 789.09) | 1832166 (1648014 to 2017386) | 634.21 (576.97 to 688.87) | -0.9 (-0.94 to -0.87) |
| South Asia - WB | 4783144 (4405110 to 5039512) | 650.85 (593.54 to 688.78) | 8341791 (7771715 to 8935786) | 612.86 (573.87 to 655.19) | -0.12 (-0.25 to 0.01) |
| World Bank Regions | 31225720 (30555208 to 31981838) | 678.59 (664.6 to 694.03) | 43704031 (41781399 to 45817178) | 529.56 (506.49 to 554.44) | -1.24 (-1.3 to -1.18) |

​

# Table S5. Spearman Correlation Results

| Test | Variables | Correlation | *P* value | Sample size | Method | Alternative |
| --- | --- | --- | --- | --- | --- | --- |
| Spearman's rank correlation | mean_sci vs gini | -1 | 0.0004 | 7 | Spearman's rank correlation rho | two.sided |

# Table S6. Results of the Multi-Group Oaxaca-Blinder Decomposition (1).​

| Comparison | Total_Difference | Endowment | Coefficient | Interaction | Endowment_Pct | Coefficient_Pct | Interaction_Pct |
| --- | --- | --- | --- | --- | --- | --- | --- |
| High income vs Low income | 1 | -0.47 | -0.3 | 0.53 | -46.62 | -29.85 | 53.26 |
| High income vs Lower middle income | 0.99 | 0.35 | 0.13 | -0.3 | 35.81 | 13.06 | -30.16 |
| High income vs Upper middle income | 0.42 | 0.07 | 0 | -0.05 | 15.66 | 1.15 | -10.7 |

# Table S7. **Results of the Multi-Group Oaxaca-Blinder Decomposition (2).**

| Comparison | Component | Percentage |
| --- | --- | --- |
| High income vs Low income | Resource Endowment Differences | -46.62 |
| High income vs Low income | Return Rate Differences | -29.85 |
| High income vs Low income | Interaction Effects | 53.26 |
| High income vs Lower middle income | Resource Endowment Differences | 35.81 |
| High income vs Lower middle income | Return Rate Differences | 13.06 |
| High income vs Lower middle income | Interaction Effects | -30.16 |
| High income vs Upper middle income | Resource Endowment Differences | 15.66 |
| High income vs Upper middle income | Return Rate Differences | 1.15 |
| High income vs Upper middle income | Interaction Effects | -10.7 |

​

# Table S8. Contribution of Various Factors to the Explained Component in Oaxaca-Blinder Decomposition

| Comparison | Resource | Percentage |
| --- | --- | --- |
| High income vs Low income | Doctors_Pct | 145.65 |
| High income vs Low income | HealthExp_Pct | -45.65 |
| High income vs Lower middle income | Doctors_Pct | 42.86 |
| High income vs Lower middle income | HealthExp_Pct | 57.14 |
| High income vs Upper middle income | Doctors_Pct | -16.67 |
| High income vs Upper middle income | HealthExp_Pct | 116.67 |

# Table S9. Cross-national Inequality Ranking by Gini Coefficient

| Iso3 | Country | Gini | Income group | Gini rank | Gini rank percent | Iso3 | Country | Gini | Income group | Gini rank | Gini rank percent |
| --- | --- | --- | --- | --- | --- | --- | --- | --- | --- | --- | --- |
| SLE | Sierra Leone | 0.1 | Low income | 1 | 0 | IRN | Iran | 0.03 | Upper middle income | 24 | 0.44 |
| MDG | Madagascar | 0.09 | Low income | 2 | 0.04 | BLR | Belarus | 0.03 | Upper middle income | 25 | 0.46 |
| MWI | Malawi | 0.08 | Low income | 3 | 0.08 | UKR | Ukraine | 0.03 | Upper middle income | 26 | 0.48 |
| NER | Niger | 0.07 | Low income | 4 | 0.13 | MUS | Mauritius | 0.03 | Upper middle income | 27 | 0.5 |
| BFA | Burkina Faso | 0.07 | Low income | 5 | 0.17 | GNQ | Equatorial Guinea | 0.03 | Upper middle income | 28 | 0.52 |
| RWA | Rwanda | 0.07 | Low income | 6 | 0.21 | MYS | Malaysia | 0.03 | Upper middle income | 29 | 0.54 |
| MOZ | Mozambique | 0.06 | Low income | 7 | 0.25 | KAZ | Kazakhstan | 0.02 | Upper middle income | 30 | 0.56 |
| TGO | Togo | 0.06 | Low income | 8 | 0.29 | ECU | Ecuador | 0.02 | Upper middle income | 31 | 0.58 |
| UGA | Uganda | 0.06 | Low income | 9 | 0.33 | BLZ | Belize | 0.02 | Upper middle income | 32 | 0.6 |
| SOM | Somalia | 0.06 | Low income | 10 | 0.38 | GEO | Georgia | 0.02 | Upper middle income | 33 | 0.62 |
| SSD | South Sudan | 0.06 | Low income | 11 | 0.42 | IRQ | Iraq | 0.02 | Upper middle income | 34 | 0.63 |
| BDI | Burundi | 0.06 | Low income | 12 | 0.46 | DOM | Dominican Republic | 0.02 | Upper middle income | 35 | 0.65 |
| GNB | Guinea-Bissau | 0.06 | Low income | 13 | 0.5 | DZA | Algeria | 0.02 | Upper middle income | 36 | 0.67 |
| ERI | Eritrea | 0.06 | Low income | 14 | 0.54 | ARG | Argentina | 0.02 | Upper middle income | 37 | 0.69 |
| GMB | Gambia | 0.06 | Low income | 15 | 0.58 | MHL | Marshall Islands | 0.02 | Upper middle income | 38 | 0.71 |
| COD | Congo - Kinshasa | 0.06 | Low income | 16 | 0.63 | TKM | Turkmenistan | 0.02 | Upper middle income | 39 | 0.73 |
| TCD | Chad | 0.05 | Low income | 17 | 0.67 | TUR | Turkey | 0.02 | Upper middle income | 40 | 0.75 |
| MLI | Mali | 0.05 | Low income | 18 | 0.71 | VCT | St. Vincent & Grenadines | 0.02 | Upper middle income | 41 | 0.77 |
| SDN | Sudan | 0.05 | Low income | 19 | 0.75 | MNG | Mongolia | 0.02 | Upper middle income | 42 | 0.79 |
| AFG | Afghanistan | 0.04 | Low income | 20 | 0.79 | JAM | Jamaica | 0.02 | Upper middle income | 43 | 0.81 |
| PRK | North Korea | 0.04 | Low income | 21 | 0.83 | TUV | Tuvalu | 0.01 | Upper middle income | 44 | 0.83 |
| SYR | Syria | 0.02 | Low income | 25 | 1 | ARM | Armenia | 0.01 | Upper middle income | 45 | 0.85 |
| FSM | Micronesia (Federated States of) | 0.21 | Lower middle income | 1 | 0 | GRD | Grenada | 0.01 | Upper middle income | 46 | 0.87 |
| MMR | Myanmar (Burma) | 0.13 | Lower middle income | 2 | 0.02 | CHN | China | 0.01 | Upper middle income | 47 | 0.88 |
| NPL | Nepal | 0.13 | Lower middle income | 3 | 0.04 | MDA | Moldova | 0.01 | Upper middle income | 48 | 0.9 |
| KIR | Kiribati | 0.1 | Lower middle income | 4 | 0.06 | LCA | St. Lucia | 0.01 | Upper middle income | 49 | 0.92 |
| IND | India | 0.1 | Lower middle income | 5 | 0.08 | LBY | Libya | 0.01 | Upper middle income | 50 | 0.94 |
| LAO | Laos | 0.08 | Lower middle income | 6 | 0.1 | WSM | Samoa | 0.01 | Upper middle income | 51 | 0.96 |
| COM | Comoros | 0.07 | Lower middle income | 7 | 0.13 | AZE | Azerbaijan | 0.01 | Upper middle income | 52 | 0.98 |
| TZA | Tanzania | 0.07 | Lower middle income | 8 | 0.15 | DMA | Dominica | 0 | Upper middle income | 53 | 1 |
| NGA | Nigeria | 0.07 | Lower middle income | 9 | 0.17 | NRU | Nauru | 0.09 | High income | 1 | 0 |
| TLS | Timor-Leste | 0.07 | Lower middle income | 10 | 0.19 | GRC | Greece | 0.07 | High income | 2 | 0.02 |
| DJI | Djibouti | 0.07 | Lower middle income | 11 | 0.21 | AUT | Austria | 0.07 | High income | 3 | 0.03 |
| CIV | Cote d'Ivoire | 0.07 | Lower middle income | 12 | 0.23 | NOR | Norway | 0.06 | High income | 4 | 0.05 |
| KHM | Cambodia | 0.07 | Lower middle income | 13 | 0.25 | SWE | Sweden | 0.05 | High income | 5 | 0.06 |
| BGD | Bangladesh | 0.06 | Lower middle income | 14 | 0.27 | KOR | South Korea | 0.05 | High income | 6 | 0.08 |
| GIN | Guinea | 0.06 | Lower middle income | 15 | 0.29 | POL | Poland | 0.05 | High income | 7 | 0.1 |
| VNM | Vietnam | 0.06 | Lower middle income | 16 | 0.31 | CHL | Chile | 0.05 | High income | 8 | 0.11 |
| ZMB | Zambia | 0.06 | Lower middle income | 17 | 0.33 | CRI | Costa Rica | 0.05 | High income | 9 | 0.13 |
| MRT | Mauritania | 0.06 | Lower middle income | 18 | 0.35 | NLD | Netherlands | 0.04 | High income | 10 | 0.15 |
| GHA | Ghana | 0.06 | Lower middle income | 19 | 0.38 | GBR | United Kingdom | 0.04 | High income | 11 | 0.16 |
| SEN | Senegal | 0.06 | Lower middle income | 20 | 0.4 | JPN | Japan | 0.04 | High income | 12 | 0.18 |
| BEN | Benin | 0.05 | Lower middle income | 21 | 0.42 | ESP | Spain | 0.04 | High income | 13 | 0.19 |
| ZWE | Zimbabwe | 0.05 | Lower middle income | 22 | 0.44 | IRL | Ireland | 0.04 | High income | 14 | 0.21 |
| VUT | Vanuatu | 0.05 | Lower middle income | 23 | 0.46 | MLT | Malta | 0.04 | High income | 15 | 0.23 |
| KEN | Kenya | 0.05 | Lower middle income | 24 | 0.48 | DEU | Germany | 0.04 | High income | 16 | 0.24 |
| LSO | Lesotho | 0.05 | Lower middle income | 25 | 0.5 | ARE | United Arab Emirates | 0.04 | High income | 17 | 0.26 |
| PNG | Papua New Guinea | 0.05 | Lower middle income | 26 | 0.52 | EST | Estonia | 0.04 | High income | 18 | 0.27 |
| PHL | Philippines | 0.05 | Lower middle income | 27 | 0.54 | ISL | Iceland | 0.04 | High income | 19 | 0.29 |
| STP | Sao Tome and Principe | 0.05 | Lower middle income | 28 | 0.56 | LUX | Luxembourg | 0.04 | High income | 20 | 0.31 |
| NAM | Namibia | 0.05 | Lower middle income | 29 | 0.58 | CAN | Canada | 0.04 | High income | 21 | 0.32 |
| COG | Congo - Brazzaville | 0.04 | Lower middle income | 30 | 0.6 | FIN | Finland | 0.04 | High income | 22 | 0.34 |
| BOL | Bolivia | 0.04 | Lower middle income | 31 | 0.63 | ITA | Italy | 0.04 | High income | 23 | 0.35 |
| SWZ | Eswatini | 0.04 | Lower middle income | 32 | 0.65 | BGR | Bulgaria | 0.04 | High income | 24 | 0.37 |
| AGO | Angola | 0.04 | Lower middle income | 33 | 0.67 | PAN | Panama | 0.03 | High income | 25 | 0.39 |
| TUN | Tunisia | 0.03 | Lower middle income | 34 | 0.69 | CYP | Cyprus | 0.03 | High income | 26 | 0.4 |
| CMR | Cameroon | 0.03 | Lower middle income | 35 | 0.71 | SYC | Seychelles | 0.03 | High income | 27 | 0.42 |
| HND | Honduras | 0.03 | Lower middle income | 36 | 0.73 | SGP | Singapore | 0.03 | High income | 28 | 0.44 |
| NIC | Nicaragua | 0.03 | Lower middle income | 37 | 0.75 | DNK | Denmark | 0.03 | High income | 29 | 0.45 |
| BTN | Bhutan | 0.03 | Lower middle income | 38 | 0.77 | ISR | Israel | 0.03 | High income | 30 | 0.47 |
| LKA | Sri Lanka | 0.03 | Lower middle income | 39 | 0.79 | MCO | Monaco | 0.03 | High income | 31 | 0.48 |
| MAR | Morocco | 0.02 | Lower middle income | 40 | 0.81 | SMR | San Marino | 0.03 | High income | 32 | 0.5 |
| PAK | Pakistan | 0.02 | Lower middle income | 41 | 0.83 | AND | Andorra | 0.03 | High income | 33 | 0.52 |
| HTI | Haiti | 0.02 | Lower middle income | 42 | 0.85 | ROU | Romania | 0.03 | High income | 34 | 0.53 |
| KGZ | Kyrgyzstan | 0.01 | Lower middle income | 43 | 0.88 | URY | Uruguay | 0.03 | High income | 35 | 0.55 |
| EGY | Egypt | 0.01 | Lower middle income | 44 | 0.9 | GUY | Guyana | 0.03 | High income | 36 | 0.56 |
| SLB | Solomon Islands | 0.01 | Lower middle income | 45 | 0.92 | BRN | Brunei | 0.03 | High income | 37 | 0.58 |
| TJK | Tajikistan | 0.01 | Lower middle income | 46 | 0.94 | LTU | Lithuania | 0.03 | High income | 38 | 0.6 |
| LBN | Lebanon | 0.01 | Lower middle income | 47 | 0.96 | NZL | New Zealand | 0.03 | High income | 39 | 0.61 |
| UZB | Uzbekistan | 0.01 | Lower middle income | 48 | 0.98 | BEL | Belgium | 0.03 | High income | 40 | 0.63 |
| JOR | Jordan | 0 | Lower middle income | 49 | 1 | HUN | Hungary | 0.03 | High income | 41 | 0.65 |
| TON | Tonga | 0.11 | Upper middle income | 1 | 0 | PLW | Palau | 0.03 | High income | 42 | 0.66 |
| GTM | Guatemala | 0.06 | Upper middle income | 2 | 0.02 | LVA | Latvia | 0.02 | High income | 43 | 0.68 |
| MDV | Maldives | 0.06 | Upper middle income | 3 | 0.04 | PRT | Portugal | 0.02 | High income | 44 | 0.69 |
| FJI | Fiji | 0.05 | Upper middle income | 4 | 0.06 | CZE | Czechia | 0.02 | High income | 45 | 0.71 |
| PER | Peru | 0.05 | Upper middle income | 5 | 0.08 | CHE | Switzerland | 0.02 | High income | 46 | 0.73 |
| BWA | Botswana | 0.05 | Upper middle income | 6 | 0.1 | QAT | Qatar | 0.02 | High income | 47 | 0.74 |
| IDN | Indonesia | 0.05 | Upper middle income | 7 | 0.12 | FRA | France | 0.02 | High income | 48 | 0.76 |
| MEX | Mexico | 0.05 | Upper middle income | 8 | 0.13 | BHR | Bahrain | 0.02 | High income | 49 | 0.77 |
| BIH | Bosnia & Herzegovina | 0.05 | Upper middle income | 9 | 0.15 | SVN | Slovenia | 0.02 | High income | 50 | 0.79 |
| ALB | Albania | 0.04 | Upper middle income | 10 | 0.17 | OMN | Oman | 0.02 | High income | 51 | 0.81 |
| THA | Thailand | 0.04 | Upper middle income | 11 | 0.19 | RUS | Russia | 0.02 | High income | 52 | 0.82 |
| CUB | Cuba | 0.04 | Upper middle income | 12 | 0.21 | AUS | Australia | 0.02 | High income | 53 | 0.84 |
| PRY | Paraguay | 0.04 | Upper middle income | 13 | 0.23 | SAU | Saudi Arabia | 0.01 | High income | 54 | 0.85 |
| ZAF | South Africa | 0.04 | Upper middle income | 14 | 0.25 | HRV | Croatia | 0.01 | High income | 55 | 0.87 |
| CPV | Cape Verde | 0.04 | Upper middle income | 15 | 0.27 | SVK | Slovakia | 0.01 | High income | 56 | 0.89 |
| GAB | Gabon | 0.04 | Upper middle income | 16 | 0.29 | KNA | St. Kitts & Nevis | 0.01 | High income | 57 | 0.9 |
| COL | Colombia | 0.04 | Upper middle income | 17 | 0.31 | BHS | Bahamas | 0.01 | High income | 58 | 0.92 |
| MNE | Montenegro | 0.04 | Upper middle income | 18 | 0.33 | USA | United States | 0.01 | High income | 59 | 0.94 |
| SRB | Serbia | 0.03 | Upper middle income | 19 | 0.35 | BRB | Barbados | 0.01 | High income | 60 | 0.95 |
| SUR | Suriname | 0.03 | Upper middle income | 20 | 0.37 | ATG | Antigua & Barbuda | 0.01 | High income | 61 | 0.97 |
| SLV | El Salvador | 0.03 | Upper middle income | 21 | 0.38 | KWT | Kuwait | 0.01 | High income | 62 | 0.98 |
| BRA | Brazil | 0.03 | Upper middle income | 22 | 0.4 | TTO | Trinidad & Tobago | 0 | High income | 63 | 1 |
| MKD | North Macedonia | 0.03 | Upper middle income | 23 | 0.42 |  | | | | | |

# Table S10. Cross-national Inequality in Gini Coefficient by Income Group

| Income group | Country | Gini | Gini rank |
| --- | --- | --- | --- |
| Low income | Sierra Leone | 0.1 | 1 |
| Lower middle income | Micronesia (Federated States of) | 0.21 | 1 |
| Upper middle income | Tonga | 0.11 | 1 |
| High income | Nauru | 0.1 | 1 |

# F**ig S1. Figure of the Basic Two-Way Fixed Effects Model Test**

Fig S1 legend: Normal Q-Q Plot (a), Residual Distribution Histogram(b), Residuals vs. Fitted Plot(c), Residual Auto-correlation Plot (d)

**[Fig S1.pdf](https://www.kdocs.cn/l/chCyEUwnfN4B)**

# F**ig S2. 2000-2021, Global NCD-SCI Inequality Trend Test**

**[Fig S2.pdf](https://www.kdocs.cn/l/cdRmQ6e7spgg)**
