# Supplementary figures and images for: Cross-national disparities in non-communicable disease: a universal health coverage-based service coverage index perspective, 2000–2021
Source: Front Public Health. 2026 Feb 10;14:1756485. doi: 10.3389/fpubh.2026.1756485 (PMC12929431; doi:10.3389/fpubh.2026.1756485)

a.

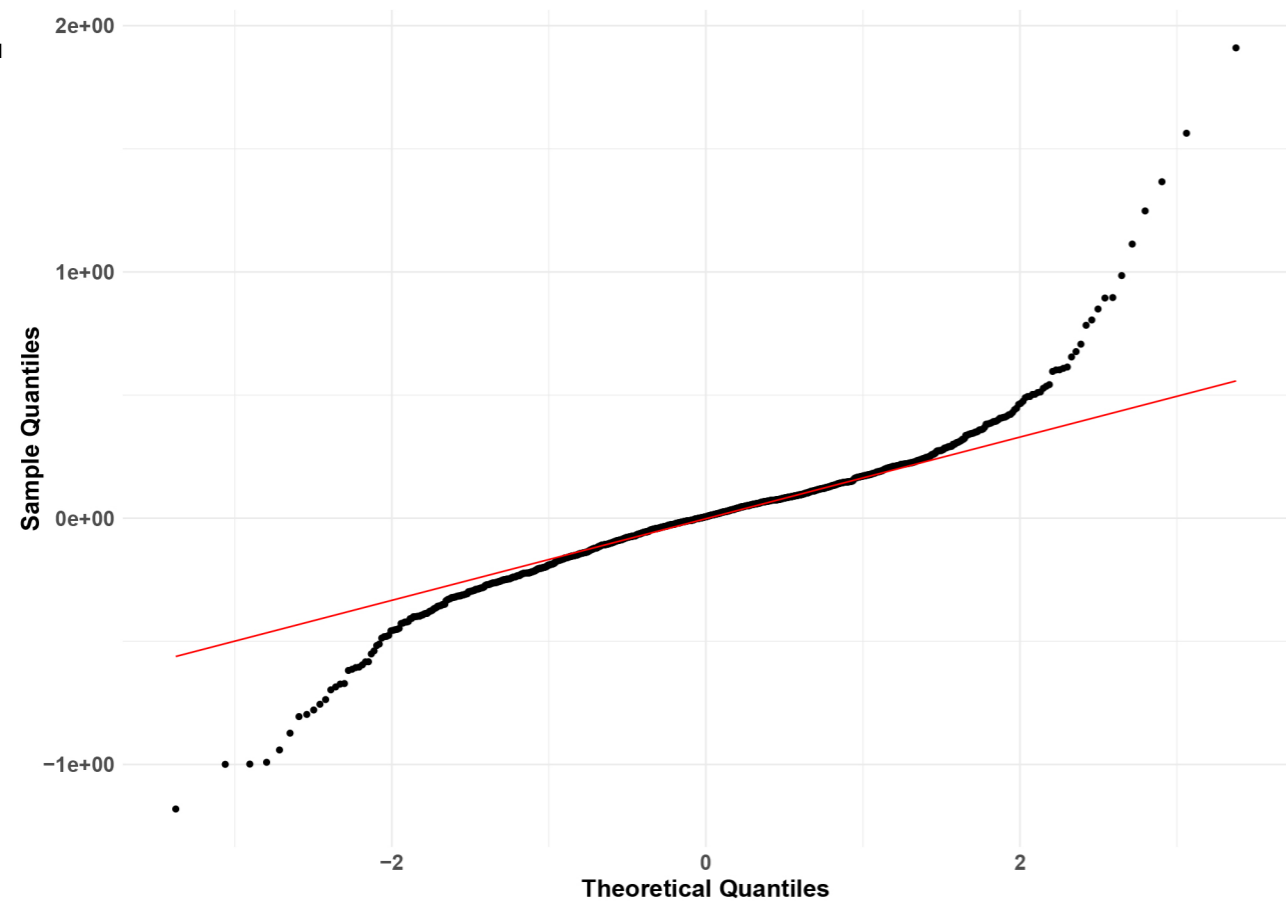

b.

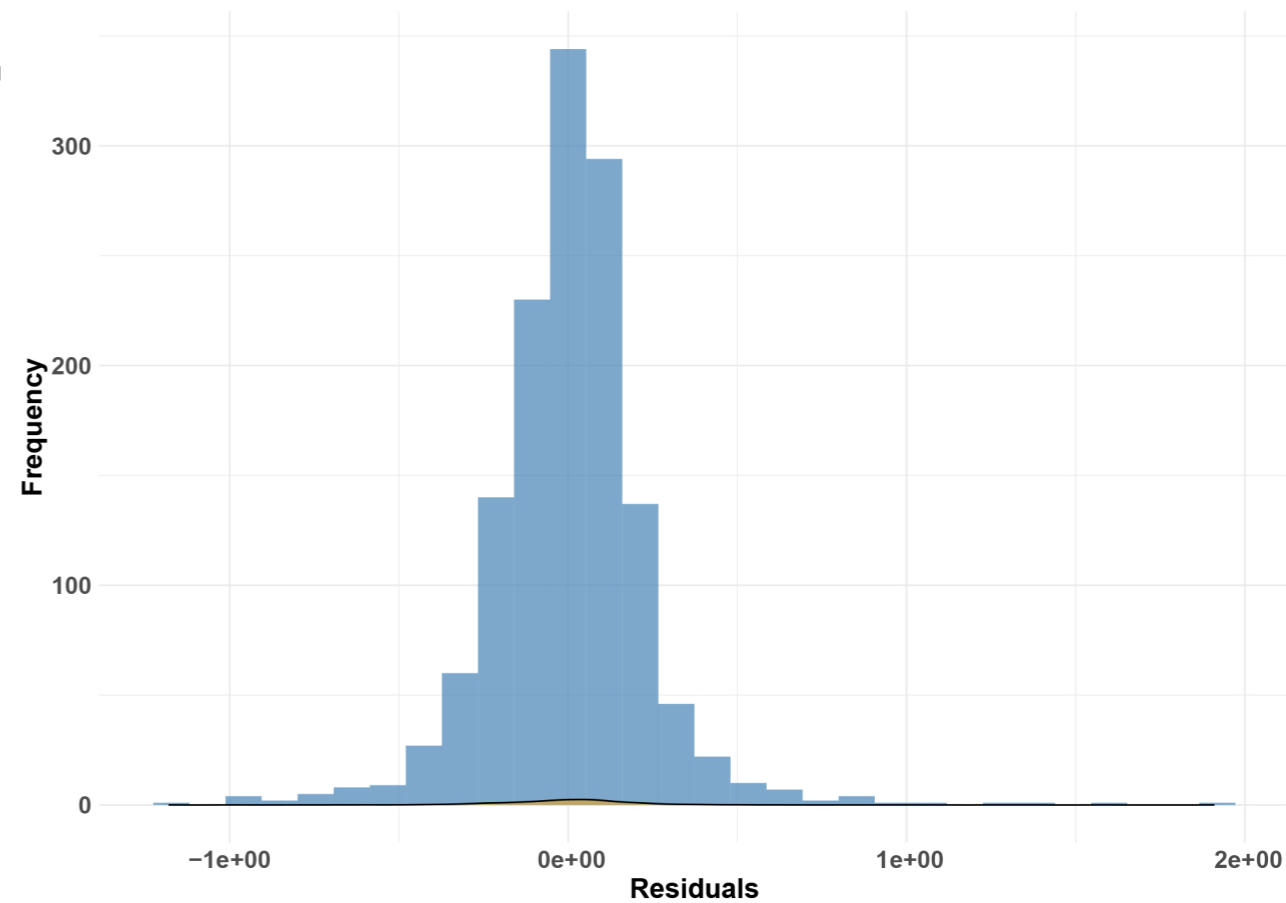

c.

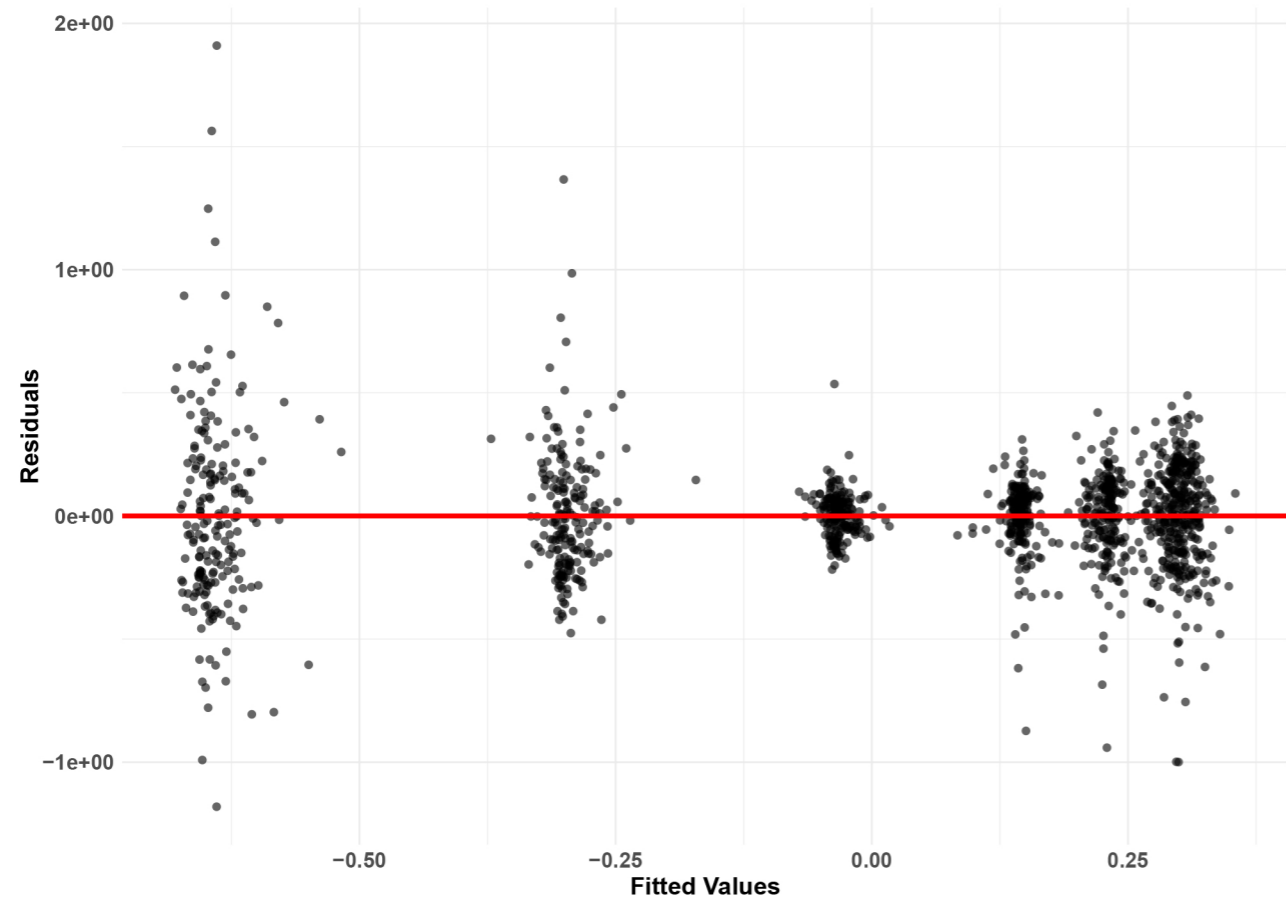

d.

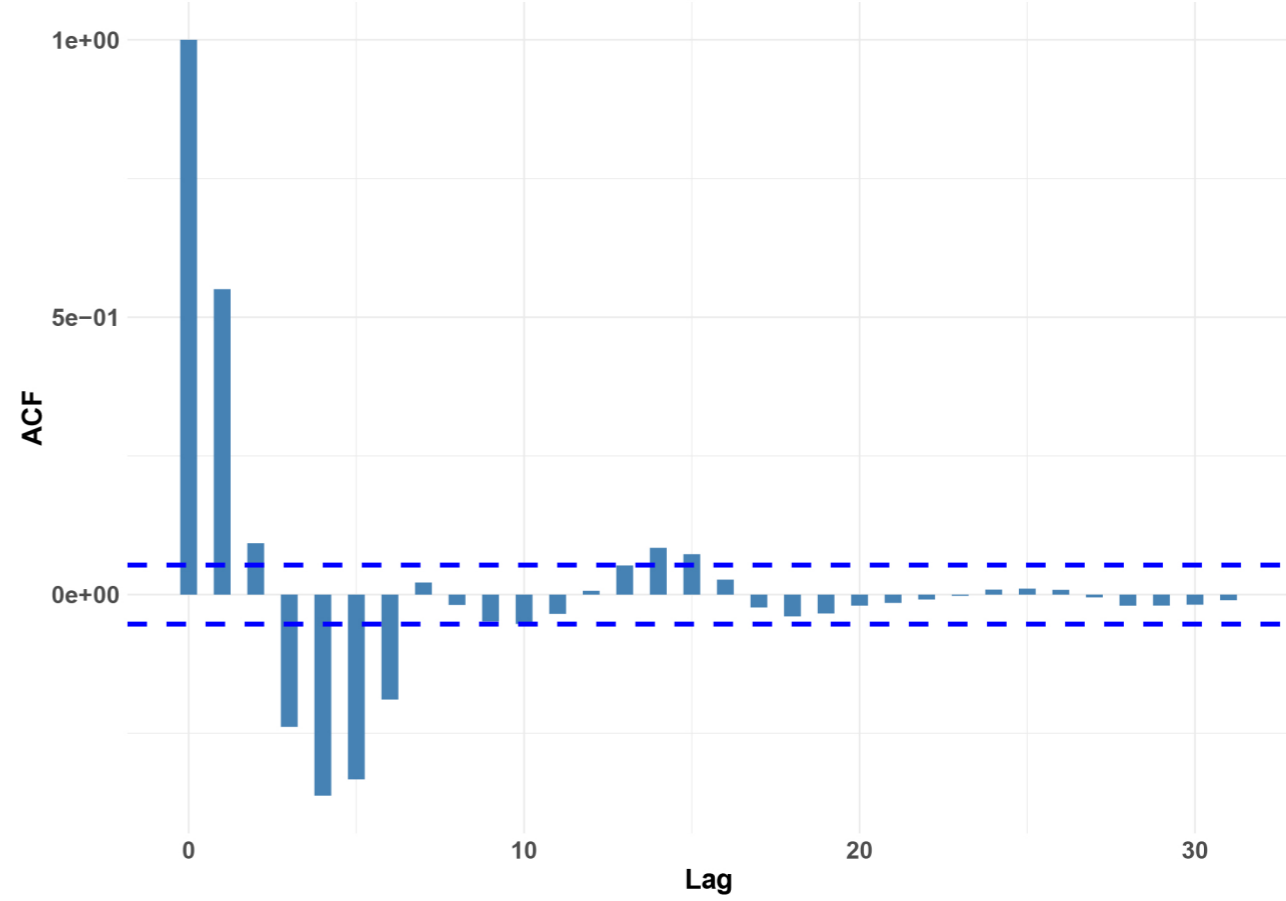

Supplement: Supplementary file 2 [file Image_1.pdf]

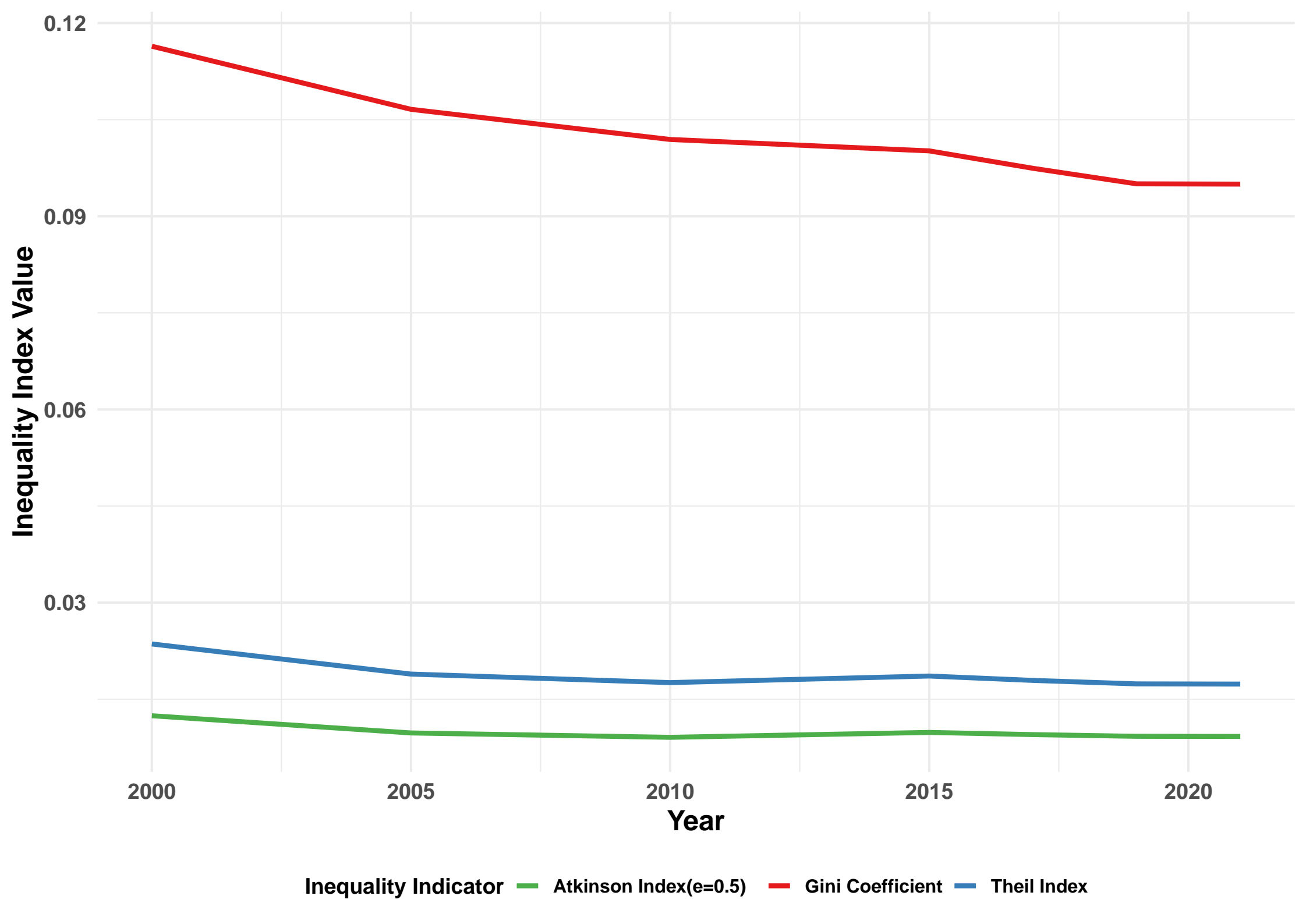

Supplement: Supplementary file 3 [file Image_2.pdf]
